# Late application of hyperbaric oxygen therapy during the rehabilitation of a patient with severe cognitive impairment after a traumatic brain injury

**DOI:** 10.1002/ccr3.3658

**Published:** 2020-12-29

**Authors:** Małgorzata Skiba, Anna Rękas‐Dudziak, Artur Bekała, Włodzimierz Płotek

**Affiliations:** ^1^ Stefan Cardinal Wyszyński District Specialist Hospital Lublin Poland; ^2^ Gynaecological‐Obstetric Clinical Hospital of the Poznań University of Medical Sciences Poznań Poland

**Keywords:** cognitive functioning, hyperbaric oxygen therapy, traumatic brain injury

## Abstract

The hyperbaric therapy resulted in the patient's quick recovery and significantly accelerated the recovery after the brain injury.

## Graphical abstract

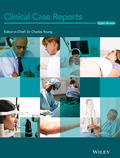

## 1 INTRODUCTION

Traumatic brain injuries (TBIs) are one of the main causes of death. TBI treatment is multidirectional, but proper brain oxygenation is one of the most important factors affecting the final result of treatment. Little is known about the possible influence of hyperbaric oxygen therapy on the function of the central nervous system (CNS) during rehabilitation after a traumatic brain injury.^1^, ^2^ The aim of the study was to analyze the case of a patient treated in a hyperbaric chamber during rehabilitation after a severe TBI. The research assumption was that exposure to an elevated pressure of oxygen may increase its availability in the CNS structures and accelerate recovery. The hyperbaric therapy resulted in the patient's quick recovery and significantly accelerated the recovery of his memory and verbal functions.

Hyperbaric oxygen therapy (HBOT) is a non‐invasive method of treating patients in a specially designed hyperbaric chamber, where they breathe 100% oxygen administered at a pressure higher than local atmospheric pressure.^3^

The use of hyperbaric oxygen in patient therapy dates back to the 17th century, when C. Henshaw, a British physician and physiologist, designed the first hyperbaric chamber.^4^ I. Boerema, a Dutch surgeon, is considered the father of modern oxygen hyperbaric oxygen therapy. It is the most effective method of supplying oxygen to all body cells, even those around which the blood supply has been disordered. During therapy, the conditions inside the hyperbaric chamber cause the blood oxygen partial pressure to increase due to a significant increase in its solubility in the plasma. Hyperbaric oxygen therapy can be successfully applied to patients with hard‐to‐heal wounds (in the course of diabetic foot syndrome, after injuries, and radiotherapy), chronic osteomyelitis, bacterial tissue infection, carbon monoxide poisoning, and extensive burns (second‐ and third‐degree burns covering over 20% of the body surface area). For therapeutic purposes, the pressure inside the chamber should exceed 1.4 atmosphere absolute (ATA) to increase the amount of oxygen supplied to cells in the body. During treatments, a pressure of 2.5 ATA is usually applied.^4^, ^5^ Thanks to hyperbaric oxygen therapy (2.0‐2.8 ATA), oxygen concentration in healthy tissues can be increased to as much as 1,000 mmHg, whereas in wounds and hypoxic areas, it can be increased up to 250 mmHg.^6^

Oxygen therapy is considered to be a safe and non‐invasive method. However, there is a risk of respiratory toxicity (Lorrain‐Smith effect: chest tightness or pain, cough, irritation or inflammation of the trachea and bronchi, apnea and reduced vital capacity, damage to the alveolar epithelium and capillary endothelium, pulmonary edema, atelectasis with hypoxia). Paul Bert effect, which may occur during HBOT, is described as a set of CNS symptoms: nausea, dizziness, hiccups, eyelid and facial tremor, vision and hearing disorders, hallucinations, breathing difficulty, fatigue, anxiety, loss of consciousness, and tonic‐clonic seizures. The most common contraindications for HBOT are as follows: pneumothorax, emphysema with CO_2_ retention, some drugs (eg, bleomycin), the presence of a pacemaker, epilepsy, fever, viral infections, spherocytosis.^7^

In recent years, this method of treatment has become more available and the list of indications where it has proved to be effective is longer. As early as 1976 there were reports on the possibility to use HBOT in CNS pathologies. It was then that Tishchenko noted that hyperbaric oxygen improved the cognitive functions and reduced the number of neurological complications in 20 patients treated in a hyperbaric chamber.^8^ Hyperbaric oxygen therapy increases the metabolism of nerve cells, reduces intracranial pressure, and improves the cognitive function and quality of life.^9^, ^10^

At the moment, the possibility of using HBOT in the late period after TBI as a method supporting rehabilitation is an issue of interest.

The aim of the study was to analyze the case of a patient after a traumatic brain injury treated with HBOT during the rehabilitation period. This type of therapy is not widely used in Poland and any case of its use may be an interesting scientific report.

## 2 CASE STUDY

Here is a case study of a 34‐year‐old patient, an academic (PhD degree) employed at a Polish university. The patient is a professional laboratory diagnostician with excellent knowledge of several foreign languages. In September 2017, he suffered a severe multiorgan injury after his motorcycle had collided with a lorry. He suffered a traumatic brain injury with accompanying epidural hematoma of the left frontal region with numerous fractures of cranial bones, including calvarial, basilar skull, and viscerocranium fractures (preliminary Glasgow Coma Scale rating: 4 points – severe TBI). After the accident, computed tomography imaging also revealed numerous bilateral rib fractures and spleen rupture. Immediately after the injury, the patient was qualified for surgery to remove the epidural hematoma and spleen. After the surgery, he was admitted to the Intensive Care Unit (ICU), where artificial ventilation was continued under analgosedation, and hyperosmotic and neuroprotective therapy was implemented. On the first day after the injury, a CT scan of the patient's head revealed significant enlargement of the area of contusion of the frontal lobes of both cerebral hemispheres. There were numerous foci of intracerebral bleeding and subarachnoid bleeding. The patient's condition improved after the therapy. After 23 days of the therapy, contact with the patient was established, but he was still suffering from sensory aphasia and significant muscle weakness. As a result of injury, the patient lost vision in his left eye. The patient stayed in the ICU for 5 months. Then he underwent rehabilitation, which continued until August 2018.

During the rehabilitation before HBOT, the patient underwent an initial neuropsychological examination, which revealed deep cognitive impairment. He could remember events only from one day or the previous 6‐9 hours. His mood was changeable and that is why he refused to take part in planned psychological tests. Attempts to conduct the Mini‐Mental State Examination ended with a few initial tasks. Frontal lobe syndrome was diagnosed.

As the patient's clinical condition was improving, he was qualified for active rehabilitation, including psychiatric rehabilitation.

As there was a chance to improve the patient's cognitive function after optimizing the supply of oxygen to the CNS, he was qualified for treatment in a hyperbaric chamber.

Within 5 months, the patient underwent a series of 42 hyperbaric oxygen therapy sessions, each of which lasted 90 minutes. During the first 3 weeks, there were sessions five times a week. Next, they were held three times a week for the next 4 weeks. The therapy was discontinued for 48 days because the patient needed to have his calvaria augmented. After the break, sessions were held three times a week for another 5 weeks.

After the therapy, the patient's nervous and mental functions as well as his motor skills and coordination improved. His memory also improved significantly, which resulted in better communication. During the therapy his cognitive processes, memory, and concentration improved. His excessive sleepiness passed away. His motor skills and vision in his left eye improved. He answered questions in full sentences. In the neuropsychologist's opinion, the patient's condition improved significantly after the hyperbaric therapy. His emotional lability disappeared and the overall level of his cognitive functions improved. His everyday communication and performance of minor chores also improved. The therapy reduced the symptoms of the frontal lobe syndrome, which was diagnosed by the neuropsychologist during the first examination.

Detailed neuropsychological assessment was possible after full HBOT. The patient maintained verbal contact but with reduced orientation to time and place. He scored low in tasks testing the course of cognitive processes. His attention and stimulus selection were disordered. He exhibited hemispatial neglect (skipped the left side of space) and visual‐spatial deficits. Unfortunately, his working memory was disordered, which resulted in a low auditory‐verbal learning level. However, the patient's direct auditory memory functioned well. The analysis of executive functions revealed organization and planning disorders, dissociation between the patient's knowledge and ability to use it, and frontal amnesia. The patient's social behavior was disordered. He exhibited verbal disinhibition, confabulation, anosognosia, reduced insight, and criticism. As far as other cognitive spheres are concerned, the patient's abstract thinking ability was reduced and he made delusional interpretations. The patient was characterized by high fatigability and despite his awareness of behavioral disorders he was not able to correct them. While staying at the rehabilitation center, the patient's state changed dynamically. His emotional lability disappeared, whereas his general level of cognitive functions and compliance with behavioral standards improved slightly. The clinical picture was dominated by visual‐spatial disorders, disorientation, and behavioral disorders, which pointed to frontal lobe syndrome. The patient's willingness to cooperate varied depending on his mood. He required permanent care. After rehabilitation with the HBOT, his short‐term memory improved, and now, he can remember the topics of conversations about 3‐4 days back.

## 3 DISCUSSION

The injury the patient suffered in the accident damaged numerous nerve structures in his body. The primary damage, which had been caused by mechanical force, stretched and disrupted his nerve cells. This condition is known as diffuse axonal injury.

Cells affected by primary injury trigger inflammatory reactions, which lead to cerebral edema and increased intracranial pressure. In addition, the resulting hematomas and inflammation, which accompanied blood extravasation, caused swelling of the adjacent tissue and aggravated dysfunction of the nervous tissue. The lesions resulting from the injury intensified the increase in the intracranial pressure, which subsequently caused a decrease in the cerebral perfusion pressure. The intracranial lesions caused the compression of undamaged vessels and reduced the flow in them. Secondary injury disordered the cerebral blood supply and resulted in the hypoxia of more distant and peripherally located parts of the brain. In the region where the perfusion of tissues is reduced, nerve cells receive too little oxygen to function properly. Therefore, their metabolism slows down and they become dormant to prevent apoptosis. This ischemic damage is potentially reversible and it can be treated by HBOT. Although indications for the HBOT in CNS disorders are optional rather than basic, this treatment method is approved by experts.^6^

Due to the increased blood oxygen content, which is maintained for a long period of time, the availability of oxygen increases and nerve cells are better oxygenated. When breathing air at atmospheric pressure, the blood oxygen tension in arterial blood is about 100 mmHg, whereas the oxygen pressure in tissues is about 55 mmHg. The increase in atmospheric pressure triples the availability of oxygen to the cells of the central nervous system. When the pressure is three times higher than atmospheric pressure and the patient is breathing pure oxygen, the blood oxygen tension increases to 2,000 mmHg, whereas the tissue oxygen tension rises up to 500 mmHg.^11^ This effect improves the oxygenation of all tissues and thus the ischemic area is reduced. This change increases cellular metabolism, which restores cellular functions disturbed during the trauma.^12^ Apart from that, hyperbaric oxygen therapy has been proved to limit post‐ischemic reduction in ATP production and to reduce the accumulation of lactates in ischemic tissues.^13^ The disruption of the mechanism that increases damage to nerve cells has a neuroprotective effect on the rest of the brain and reduces the extent of permanent damage.

Another mechanism that may significantly affect the treatment is the influence of hyperbaric oxygen therapy on vasoconstriction and vasodilatation of cerebral vessels. After exposure to hyperbaric oxygen, the cerebral blood flow is reduced due to lower concentration of nitric oxide. An experiment on rats exposed to pressures of 3 and 4 ATA for 30 minutes showed that their regional cerebral flow decreased respectively by 26%‐39% and 37%‐43% and this effect lasted up to 75 min. The effect persisted longer in the group of the animals which had received nitric oxide (N(omega)‐nitro‐L‐arginine‐methyl‐ester) prior to the exposure. In the same experiment, the nitric oxide concentration increased during further exposure and caused a secondary increase in the regional blood flow in the brains of all rats.^14^

Harch et al subjected rats to HBOT 31‐33 days after experimental cerebral contusion. The animals had 80 sessions at a pressure of 1.5 ATA. Improvement in behavioral and neurobiological outcomes was assessed in the study. The animals' blood vessel density was measured bilaterally in the hippocampus by means of diaminobenzidine staining and correlated with the results of behavioral tests. Vascular density in the damaged hippocampus increased significantly. In consequence, spatial movement in the group subjected to HBOT increased significantly, as compared with the control groups.^15^ Repeated exposure stimulates the growth of blood vessels by increasing the secretion of the vascular endothelial growth factor (VEGF) by macrophages. Experimental studies showed that the HBOT brought significantly better results in mice after brain injury, both in the cognitive and motor range.^16^

The production of oxygen free radicals stimulates anti‐inflammatory mechanisms, which later reduce cerebral edema and thus compensate for the re‐expansion of blood vessels. A study conducted on mice with induced brain injury showed that the interleukin‐10 level increased, whereas cerebral edema decreased as early as 3 hours after hyperbaric oxygen therapy at a pressure of 2 ATA.^17^ Three exposures to hyperbaric conditions at a pressure of 2 ATA reduced the inflammatory markers and increased the number of new endothelial and glial cells (Lin et al, 2012). Another study showed that after HBOT, the caspase‐3 and interleukin‐8 levels as well as the tumor necrosis factor alpha level (TNF‐α) decreased.^18^ The intensity of free radical production and lipid peroxidation was investigated in an experiment on rabbits with total brain ischemia induced for 10 minutes by infusion of artificial cerebrospinal fluid into the subarachnoid space. Next, immediately after reperfusion the test group was placed in a hyperbaric chamber at a pressure of 2.8 ATA for 75 minutes. Meanwhile, the control group breathed atmospheric air. The concentrations of oxidized and free glutathione and malondialdehyde were measured in the experiment. The neurophysiological symptoms of brain damage were assessed by analyzing the cortical somatosensory‐evoked potentials. The production of oxygen free radicals increased in the test group exposed to the hyperbaric environment, because there was a higher ratio of oxidized to reduced glutathione. Lipid peroxidation was comparable in both groups, as evidenced by the malondialdehyde level. The somatosensory evoked potentials were as much as 50% higher in the group of rabbits subjected to hyperbaric oxygen therapy.^19^

The publications discussed above described laboratory tests on animals and the period directly related to the moment of TBI. It is extremely difficult to use HBOT in humans in the immediate period after TBI. The problem of HBOT efficacy in people in the late period following damage to the central nervous system (CNS) should be carefully evaluated, because to date, there have been few studies describing the problem.

Efrata et al described the beneficial effects of HBOT applied in the neurological rehabilitation of 74 patients after stroke. They had 40 HBOT sessions at a pressure of 2 ATM for 2 months. The hyperbaric treatment improved the patients' neurological functions, including speech, more than the standard treatment applied to other patients.^20^ The patient described in our case study had TBI rather than acute CNS ischemia, but in TBI pathogenesis massive blood supply disorders are an important link in the CNS pathology chain, so the possible positive effect of HBOT in TBI can also be broadly taken into consideration. In our case, the patient had a similar number of HBOT sessions.

The following areas are particularly vulnerable in TBI: the frontal area, the subfrontal white matter, the deeper midline structures including the basal ganglia and diencephalon, the rostral brain stem, and the temporal lobes including the hippocampi. In the course of TBI the catecholaminergic and cholinergic relay systems, which are involved in the regulation of arousal, cognition, reward behavior and mood, are particularly vulnerable. Damage to the dorsolateral prefrontal cortex impairs executive functions. The orbitofrontal cortex is responsible for intuitive social behaviors. The third important system is the neuronal system related to the anterior cingulate cortex, which is involved in reward‐related behaviors.^21^ Imaging tests conducted on our patient revealed structural damage to similar regions, which was reflected by his cognitive status. During the HBOT, the patient's cognitive functions and behavior improved significantly.

Golden et al made a statistical analysis of 50 patients after TBI who underwent SPECT before, during, and after hyperbaric oxygen therapy. The results of this analysis confirmed the hypothesis that HBOT improved blood supply in the cortex, while the therapy had no effect on the region of the pons and cerebellum. The blood supply was better in younger patients, but the improvement in functions was comparable in both groups.^22^ In the context of the research conducted by Golden et al, it was justified to apply HBOT to our patient due to his young age and the fact that traumatic lesions were mostly located in his cerebral cortex. Boussi‐Gross observed that treatment in a hyperbaric chamber improved the quality of life of patients after TBI. The researcher suggested that neuroplasticity played a role in improvement of chronically impaired brain functions.^23^

Hadanny et al described the use of HBOT in a distant time after brain damage. The study was conducted on patients who had suffered brain injury 3 months to 33 years before. A team of scientists observed significant improvement in cognitive functions in correlation with an increase in the neurological activity in individual parts of the brain. After the HBOT, the patients' memory and attention usually improved.^24^

In our case, the patient was qualified for HBOT due to the persistence of severe cognitive deficit. He was qualified for the treatment with due caution. The patient did not develop epilepsy, which might have disqualified him from therapy. The potential pathogenic effect of the concomitant chest injury was also taken into consideration. However, the patient did not develop pneumothorax despite numerous rib fractures. As the time interval between the injury and HBOT allowed full recovery from respiratory pathologies, there was low risk of lung damage during the HBOT.

The case described in this article documents the effectiveness of using HBOT to treat the patient after a severe TBI injury complicated by significant sensory aphasia. Significant neurological complications can be expected after such a severe injury, because there is a linear correlation between the GCS score and the occurrence of severe neurological disorders within a GCS range of 3‐9.^25^

It is disputable whether the observed neurological improvement resulted from the natural course of the disease or it was accelerated by the HBOT.^26^ According to reference publications, the language disorder tends to disappear naturally within 1‐3 months.^27^ As our patient had severe cognitive impairment after 5 months of ITU treatment, we can assume that the changes regressed extremely slowly and the risk of chronic cognitive impairment was high. In the context of the aforementioned reports, we can hypothesize that the HBOT had beneficial effect on our patient. According to recent reports, treatment in a hyperbaric chamber is safe and beneficial to patients after a traumatic brain injury and those with symptoms of post‐traumatic stress disorder and post‐concussion syndrome.^28^

Another aspect to be taken into consideration is the patient's higher cognitive level before the TBI. According to the cognitive reserve theory, patients with initially higher IQ and higher level of education function cognitively better after TBI. Kesler et al compared the total intracranial volume (TICV) and ventricle‐to‐brain ratio (VBR) by means of high‐resolution magnetic resonance imaging.^29^, ^30^ They also analyzed the level of education and used standardized tests to compare the cognitive outcome of 25 patients before and after TBI. The results of this study suggest that a larger premorbid brain volume and a higher level of education may decrease vulnerability to cognitive deficits following TBI, which is consistent with the cognitive reserve concept.^31^ There was an analogous situation in our study, because HBOT was applied to the patient with a high initial level of education (patient's IQ before TBI unknown). Therefore, the patient's high cognitive reserve may have influenced the positive outcome of HBOT.

Currently, there are only 12 hyperbaric oxygen therapy centers in Poland, which are mostly located in large medical centers. Therefore, there are limited possibilities to apply this therapy in common TBI cases. Additional experience in optional HBOT uses may be a source of important information broadening our knowledge and the scope of therapy applied to our patients. As the awareness of healthcare workers concerning this therapeutic option in TBI is increasing, the application of HBOT may extend and result in secondary assessment of its effectiveness in patients with CNS pathology.

In the future studies, comparing the results of rehabilitation with various HBOT schemes may be the basis for modification and extension of the current treatment scheme for patients with TBI.

## 4 CONCLUSION

The use of HBOT in the course of rehabilitation was safe for the patient after TBI and it may have shortened the recovery of neurological functions. Further research is necessary to precisely determine the influence of HBOT on the recovery process.

## CONFLICT OF INTEREST

In behalf of My and Co‐authors, I certify that there is no actual or potential conflict of interest in relation to this article.

## AUTHOR CONTRIBUTIONS

ARD, MS, AB and WP: contributed to the design and implementation of the case report, to the analysis of the results and to the writing of the manuscript.

## CONSENT FOR PUBLICATION

The consent to the publication of the data was issued by the Bioethics Committee at the Medical University of Lublin based on the written and oral consent of the patient to the publication of data on 10/11/2019.

## Data Availability

The data that support the findings of this study are available from the corresponding author upon reasonable request.

## References

[1] Adamides AA , Winter CD , Lewis PM , Cooper DJ , Kossmann T . Current controversies in the management of patients with severe traumatic brain injury. ANZ J Surg. 2006;76:163‐174. 16626360 10.1111/j.1445-2197.2006.03674.x

[2] Boerema I , Meyne N , Brum‐Melkamp W . Life without blood: a study of the influence of high atmospheric pressure and hypothermia on dilution of the blood. J Cardiovasc Surg. 1960;1:133‐146.

[3] Narożny W , Siebert J . Możliwości i ograniczenia stosowania hiperbarii tlenowej w medycynie. (The Possibilities and Limitations to the Use of Hyperbaric Oxygen Therapy in Medicine). Forum Medycyny Rodzinnej. 2007;1:368‐375.

[4] Jain KK . Textbook of hyperbaric medicine, 4th edn Göttingen: Hogrefe & Huber Publishers; 2004.

[5] Mathieu D . Handbook on hyperbaric medicine. Dordecht: Springer; 2006.

[6] Knefel G . Podstawy hiperbarycznej terapii tlenowej (The Essentials of Hyperbaric Oxygen Therapy). Leczenie Ran. 2006;3:83‐93.

[7] Szymańska B , Kawecki M , Knefel G . Kliniczne aspekty hiperbarii tlenowej (Clinical Aspects of Hyperbaric Oxygen Therapy). Wiadomości Lekarskie, LIX. 2006;59(1‐2):105‐109. 16646303

[8] Tishchenko AT . Hyperbaric oxygen therapy in the clinical treatment of mental disorders accompanying severe cranio‐cerebral trauma. Zh Nevropatol Psikhiatr Im S S Korsakova. 1976;76:262‐268. 1266492

[9] Deng Z , Chen W , Jin J , Zhao J , Xu H . The neuroprotection effect of oxygen therapy: a systematic review and meta‐analysis. Nigerian J Clin Pract. 2018;21:401‐416. 10.4103/njcp.njcp_315_16 29607850

[10] Rockswold SB , Rockswold GL , Zaun DA , et al. A prospective, randomized clinical trial to compare the effect of hyperbaric to normobaric hyperoxia on cerebral metabolism, intracranial pressure, and oxygen toxicity in severe traumatic brain injury. J Neurosurg. 2010;112:1080‐1094. 19852540 10.3171/2009.7.JNS09363

[11] Tibbles PM , Edelsberg JS . Hyperbaric oxygen therapy. N Engl J Med. 1996;334:1642‐1648. 8628361 10.1056/NEJM199606203342506

[12] Daugherty WP , Levasseur JE , Sun D , Rocksworld GL , Bullock MR . Effects of hyperbolic oxygen therapy on cerebral oxygenation and mitochondrial function following moderate lateral fluid‐percussion injury in rats. J Neurosurg. 2004;101:499‐504. 15352608 10.3171/jns.2004.101.3.0499

[13] Stewart RJ , Yamaguchi KT , Mason SW , Roshdieh BB , Dabassi NI , Ness NT . Tissue ATP levels in burn injured skin treated with hyperbaric oxygenation. Undersea Biomed Res. 1989;16(Suppl):53.2648656

[14] Demchenko IT , Boso AE , O'Neill TJ , Bennett PB , Piantadosi CA . Nitric oxide and cerebral blood flow responses to hyperbaric oxygen. J Appl Physiol. 2000;88:1381‐1389. 10749833 10.1152/jappl.2000.88.4.1381

[15] Harch PG , Kredit C , Van Meter KW , Sutherland RJ . Hyperbaric oxygen therapy improves spatial learning and memory in a rat model of chronic traumatic brain injury. Brain Res. 2007;1174:120‐129. 17869230 10.1016/j.brainres.2007.06.105

[16] Beratz‐Goldstein R , Toussia‐Cohen S , Elpaz A , Rubovitch V , Pick CG . Immediate and delated hyperbaric oxygen therapy as a neuroprotective treatment for traumatic brain injury in mice. Mol Cell Neurosci. 2017;83:74‐82. 28690173 10.1016/j.mcn.2017.06.004

[17] Chen X , Duan XS , Xu LJ , Zhao JJ , She ZF . Interleukin‐10 mediates the neuroprotection of hyperbaric oxygen therapy against traumatic brain injury in mice. Neuroscience. 2014;266:235‐243. 24291771 10.1016/j.neuroscience.2013.11.036

[18] Zhang Y , Yang Y , Tang H , et al. Hyperbaric oxygen therapy ameliorates local brain metabolism, brain edema and inflammatory response in a blast‐induced traumatic brain injury model in rabbits. Neurochem Res. 2014;39:950‐960. 24682753 10.1007/s11064-014-1292-4

[19] Mink RB , Dudka AJ . Hyperbaric oxygen after global cerebral ischemia in rabbits does not promote lipid per oxidation. Crit Care Med. 1995;23:1398‐1404. 7634811 10.1097/00003246-199508000-00014

[20] Efrati S , Fishlev G , Bechor Y , Volkov O , Bergan J . Hyperbaric oxygen induces late neuroplasticity in post stroke patients–randomized, prospective trial. PLoS ONE. 2013;8(1):e53716. 23335971 10.1371/journal.pone.0053716 PMC3546039

[21] McAllister TW . Neurobiological consequences of traumatic brain injury. Dialogues Clin Neurosci. 2011;13:287‐300. 22033563 10.31887/DCNS.2011.13.2/tmcallister PMC3182015

[22] Golden ZL , Neubauer R , Golden CJ , Greene L , Marsh J , Melko A . Improvement in cerebral metabolism in chronic brain injury after hyperbaric oxygen therapy. J Neurosci. 2009;112:119‐131. 10.1080/00207450212027 12325401

[23] Boussi‐Gross R , Golan H , Fishlev G , Bechor Y , Volkov O . Hyperbaric oxygen therapy can improve post‐concussion syndrome years after mild traumatic brain injury ‐ randomized prospective trial. PLoS ONE. 2013;8(11):e79995 10.1371/journal.pone.0079995 24260334 PMC3829860

[24] Hadanny A , Abbott S , Suzin G , Bechor Y , Efrati S . Effect of hyperbaric oxygen therapy on chronic neurocognitive deficits of post‐traumatic brain injury patients: retrospective analysis. BMJ Open. 2018;8(9):e023387. 10.1136/bmjopen-2018-023387 PMC6169752 30269074

[25] Hukkelhoven CW , Rampen AJ , Maas AI , Farace E , Habbema JD . Some prognostic models for traumatic brain injury were not valid. J Clin Epidemiol. 2006;59(2):132‐143. 16426948 10.1016/j.jclinepi.2005.06.009

[26] Wang GH , Zhang XG , Jiang ZL , et al. Neuroprotective effects of hyperbaric oxygen treatment on traumatic brain injury in the rat. J Neurotrauma. 2010;27:1733‐1743. 20568957 10.1089/neu.2009.1175

[27] Wood RL .Neurobehavioral sequelae of traumatic brain injury. Taylor & Francis, 1990:98.

[28] Harch PG , Andrews SR , Forgarty EF , Lucarini J , Van Meter KW . Case control study: hyperbaric oxygen treatment of mild traumatic brain injury persistent post‐concussion syndrome and post‐traumatic stress disorder. Medical Gas Res. 2017;7:156‐174. 10.4103/2045-9912.215745 PMC5674654 29152209

[29] Cihan YB , Uzun G , Yildiz S , Dönmez H . Hyperbaric oxygen therapy for radiation‐induced brain necrosis in a patient with primary central nervous system lymphoma. J Surgical Oncology. 2009;100:732‐735. 10.1002/jso.21387 19722227

[30] Feldmeier JJ . Hyperbaric oxygen for delayed radiation injuries. Undersea Hyperb Med. 2004;31:133‐145. 15233169

[31] Kesler SR , Adams HF , Blasey CM , Bigler ED . Premorbid intellectual functioning, education, and brain size in traumatic brain injury: an investigation of the cognitive reserve hypothesis. Appl Neuropsychol. 2003;10:153‐162. 12890641 10.1207/S15324826AN1003_04

